# Triple Diamond-Alpha integral and Hölder-type inequalities

**DOI:** 10.1186/s13660-018-1704-0

**Published:** 2018-05-10

**Authors:** Jing-Feng Tian

**Affiliations:** College of Science and Technology, North China Electric Power University, Baoding, P.R. China

**Keywords:** 26D15, Hölder’s inequality, Minkowski’s inequality, Triple Diamond-Alpha integral, Time scales

## Abstract

In this paper, we first introduce the definition of triple Diamond-Alpha integral for functions of three variables. Therefore, we present the Hölder and reverse Hölder inequalities for the triple Diamond-Alpha integral on time scales, and then we obtain some new generalizations of the Hölder and reverse Hölder inequalities for the triple Diamond-Alpha integral. Moreover, using the obtained results, we give a new generalization of the Minkowski inequality for the triple Diamond-Alpha integral on time scales.

## Introduction

To unify and generalize discrete and continuous analysis, in 1998, Hilger [1] introduced the theory of time scales. Since then, many researchers have studied various aspects of the theory and obtained a lot of interesting results on time scales [1–10]. The first purpose of this paper is to give the definition of the triple Diamond-Alpha integral (triple diamond-*α* integral or triple $\diamond_{\alpha}$-integral) for functions of three variables on time scales.

Let $u(x)$ and $v(x)$ be continuous real-valued functions on $[a, b]$, and let $\frac{1}{p}+ \frac{1}{q}=1$.

(I) if $p>1$ and if $u(x)\geq0$, $v(x)\geq0$, then the classical Hölder inequality holds (see [11]):
  1 $$ \int_{a}^{b}u(x)v(x)\,dx\leq \biggl( \int_{a}^{b}u^{p}(x)\,dx \biggr)^{\frac{1}{p}} \biggl( \int_{a}^{b}v^{q}(x)\,dx \biggr)^{\frac{1}{q}}. $$
(II) if $0< p<1$ and if $u(x)>0$, $v(x)>0$, then the following reverse Hölder inequality (e.g., see [12]) holds:
  2 $$ \int_{a}^{b}u(x)v(x)\,dx \geq \biggl( \int_{a}^{b}u^{p}(x)\,dx \biggr)^{\frac{1}{p}} \biggl( \int_{a}^{b}v^{q}(x)\,dx \biggr)^{\frac{1}{q}}. $$

The classical Hölder and reverse Hölder inequalities play a very important role and have wide applications in different branches of modern mathematics. A large number of papers dealing with refinements, generalizations, and applications of the Hölder and reverse Hölder inequalities and their series analogues in different ares of mathematics have appeared. For example, Agahi et al. [13] gave generalizations of the Hölder and reverse Hölder inequalities for the pseudo-integral. Zhao et al. [14] found that the Hölder inequality for the pan-integral holds if the monotone measurer is subadditive. Tian [15–18] gave some new properties and refinements of the Hölder and reverse Hölder inequalities. For more detail, the reader may consult [19–25].

Among various extensions of (1) and (2), Agarwal, Bohner, and Peterson first presented the time scale versions of (1) via the Delta-integral (Δ-integral).

### Theorem A

*Assume that* $\mathbb{T}$ *is a time scale*, $a,b\in \mathbb{T}$, *and* $a< b$. *If* $p>1$ *with* $\frac{1}{p}+\frac{1}{q}=1$ *and if* $u, v\in C_{rd}([a, b], \mathbb{R})$, *then*

3 $$ \int_{a}^{b} \bigl\vert u(x)v(x) \bigr\vert \Delta x \leq \biggl( \int_{a}^{b} \bigl\vert u(x) \bigr\vert ^{p}\Delta x \biggr)^{\frac {1}{p}} \biggl( \int_{a}^{b} \bigl\vert v(x) \bigr\vert ^{q}\Delta x \biggr)^{\frac{1}{q}}. $$

Later, in 2005, Wong et al. [26] gave the following Hölder-type inequalities via the Delta-integral.

### Theorem B

*Assume that* $\mathbb{T}$ *is a time scale*, $a,b\in \mathbb{T}$, *and* $a< b$. *Let* $\frac{1}{p}+\frac{1}{q}=1$ *and* $u, v, \omega\in C_{rd}([a, b], \mathbb{R})$. *If* $p>1$, *then*

4 $$ \int_{a}^{b} \bigl\vert \omega(x) \bigr\vert \bigl\vert u(x)v(x) \bigr\vert \Delta x \leq \biggl( \int_{a}^{b} \bigl\vert \omega(x) \bigr\vert \bigl\vert u(x) \bigr\vert ^{p}\Delta x \biggr)^{\frac{1}{p}} \biggl( \int_{a}^{b} \bigl\vert \omega(x) \bigr\vert \bigl\vert v(x) \bigr\vert ^{q}\Delta x \biggr)^{\frac{1}{q}} . $$

*If* $p<0$ *or* $q<0$, *then inequality* (4) *is reversed*.

In 2008, Özkan et al. [27] presented the following time scale versions of inequalities (1) and (2) via the Nabla-integral (∇-integral) and Diamond-Alpha integral ($\diamond_{\alpha}$-integral).

### Theorem C

*Assume that* $\mathbb{T}$ *is a time scale*, $a,b\in\mathbb{T}$, *and* $a< b$. *Let* $\frac{1}{p}+\frac{1}{q}=1$ *and* $u, v, \omega\in C_{ld}([a, b], \mathbb{R})$. *If* $p>1$, *then*

5 $$ \int_{a}^{b} \bigl\vert \omega(x) \bigr\vert \bigl\vert u(x)v(x) \bigr\vert \nabla x\leq \biggl( \int_{a}^{b} \bigl\vert \omega(x) \bigr\vert \bigl\vert u(x) \bigr\vert ^{p}\nabla x \biggr)^{\frac{1}{p}} \biggl( \int_{a}^{b} \bigl\vert \omega(x) \bigr\vert \bigl\vert v(x) \bigr\vert ^{q}\nabla x \biggr)^{\frac{1}{q}}; $$

*If* $p<0$ *or* $q<0$, *then inequality* (5) *is reversed*.

### Theorem D

*Assume that* $\mathbb{T}$ *is a time scale*, $a,b\in\mathbb{T}$, *and* $a< b$. *Let* $\frac{1}{p}+\frac{1}{q}=1$, *and let* $u, v, \omega: [a, b]\rightarrow\mathbb{R}$ *be* $\diamond_{\alpha}$-*integrable functions*. *If* $p>1$, *then*

6 $$ \int_{a}^{b} \bigl\vert \omega(x) \bigr\vert \bigl\vert u(x)v(x) \bigr\vert \diamond_{\alpha} x\leq \biggl( \int_{a}^{b} \bigl\vert \omega(x) \bigr\vert \bigl\vert u(x) \bigr\vert ^{p}\diamond_{\alpha} x \biggr)^{\frac{1}{p}} \biggl( \int_{a}^{b} \bigl\vert \omega(x) \bigr\vert \bigl\vert v(x) \bigr\vert ^{q} \diamond _{\alpha}x \biggr)^{\frac{1}{q}} . $$

*If* $p<0$ *or* $q<0$, *then inequality* (6) *is reversed*.

### Remark 1.1

If $\alpha=0$ in Theorem C, then inequality (6) reduces to inequality (5). If $\alpha=1$ in Theorem C, then inequality (6) reduces to inequality (4).

The second purpose of this paper is to give the time scale versions of the Hölder and reverse Hölder inequalities for the triple Diamond-Alpha integral. Then we obtain some new generalizations of the Hölder and reverse Hölder inequalities for the triple Diamond-Alpha integral on time scales. Moreover, using the obtained results, we present a new generalization of the Minkowski inequality for the triple Diamond-Alpha integral on time scales.

## Main results

For details on time scales theory, the readers may consult [1, 3–9] and the references therein. Now we give the definition of triple Diamond-Alpha integral for functions of three variables.

The triple Diamond-Alpha integral is defined as an iterated integral. Suppose that $\mathbb{T}$ is a time scale and $a_{i}, b_{i}\in\mathbb {T}$ with $a_{i}< b_{i}$ $(i=1,2,3)$. Let $f(x_{1},x_{2},x_{3})$ be a real-valued function on $\mathbb{T}\times\mathbb{T}\times\mathbb{T}$. Because we need the notation of partial derivatives with respect to variables $x_{i}$, we denote the time scale partial derivatives of $f(x_{1},x_{2},x_{3})$ with respect to $x_{i}$ by $f^{\diamond_{\alpha}^{i}} (x_{1},x_{2},x_{3})$, $i=1,2,3$. We now give the definition of these partial derivatives. Fixing $x_{2},x_{3}\in\mathbb{T}$, the diamond-*α* derivative of a function

$$\begin{aligned} &\mathbb{T}\rightarrow\mathbb{R}, \\ &x_{1}\rightarrow f(x_{1},x_{2},x_{3}) \end{aligned}$$

is denoted by $f^{{\diamond}_{\alpha}^{1}}$. Next, fixing $x_{1},x_{3}\in\mathbb{T}$, the diamond-*α* derivative of a function

$$\begin{aligned} &\mathbb{T}\rightarrow\mathbb{R}, \\ &x_{2}\rightarrow f(x_{1},x_{2},x_{3}) \end{aligned}$$

is denoted by $f^{{\diamond}_{\alpha}^{2}}$. Finally, fixing $x_{1}, x_{2}\in\mathbb{T}$, the diamond-*α* derivative of a function

$$\begin{aligned} &\mathbb{T}\rightarrow\mathbb{R}, \\ &x_{3}\rightarrow f(x_{1},x_{2},x_{3}) \end{aligned}$$

is denoted by $f^{{\diamond}_{\alpha}^{3}}$.

If a function *f* has a $\diamond_{\alpha}^{1}$ antiderivative $F_{1}$, $F_{1}$ has a $\diamond_{\alpha}^{2}$ antiderivative $F_{2}$, and $F_{2}$ has a $\diamond_{\alpha}^{3}$ antiderivative $F_{3}$, that is, $F^{{\diamond}_{\alpha}^{1}}_{1}=f$, $F^{{\diamond}_{\alpha }^{2}}_{2}=F_{1}$, and $F^{{\diamond}_{\alpha}^{3}}_{3}=F_{2}$, then

7 $$\begin{aligned} & \int_{a_{1}}^{b_{1}} \int_{a_{2}}^{b_{2}} \int _{a_{3}}^{b_{3}}f(x_{1},x_{2},x_{3}) \diamond_{\alpha} x_{1}\diamond_{\alpha} x_{2} \diamond_{\alpha} x_{3} \\ &\quad:= \int_{a_{2}}^{b_{2}} \int_{a_{3}}^{b_{3}} \bigl(F_{1}(b_{1},x_{2},x_{3})-F_{1}(a_{1},x_{2},x_{3}) \bigr)\diamond_{\alpha} x_{2}\diamond_{\alpha} x_{3} \\ &\quad= \int_{a_{3}}^{b_{3}} \bigl[F_{2}(b_{1},b_{2},x_{3})-F_{2}(b_{1},a_{2},x_{3}) - \bigl(F_{2}(a_{1},b_{2},x_{3})-F_{2}(a_{1},a_{2},x_{3}) \bigr) \bigr]\diamond_{\alpha} x_{3} \\ &\quad=F_{3}(b_{1},b_{2},b_{3})-F_{3}(b_{1},b_{2},a_{3}) -F_{3}(b_{1},a_{2},b_{3})+F_{3}(b_{1},a_{2},a_{3}) \\ &\qquad{} -F_{3}(a_{1},b_{2},b_{3})+F_{3}(a_{1},b_{2},a_{3}) +F_{3}(a_{1},a_{2},b_{3})-F_{3}(a_{1},a_{2},a_{3}) . \end{aligned}$$

By this definition it is easy to obtain the following property for the triple Diamond-Alpha integral.

### Proposition 2.1

*Suppose that* $\mathbb{T}$ *is a time scale*, $a_{i}, b_{i}\in\mathbb{T}$ *with* $a_{i}< b_{i}$ $(i=1,2,3)$, *and* $f(x_{1},x_{2},x_{3})$ *and* $g(x_{1},x_{2},x_{3})$ *are* $\diamond_{\alpha}$-*integrable functions on* $[a_{i}, b_{i}]^{3}_{\mathbb{T}}$ $(i=1,2,3)$.

(i) *If* $f(x_{1},x_{2},x_{3})\geq0$ *for all* $x_{i}\in[a_{i}, b_{i}]_{\mathbb{T}}$ $(i=1,2,3)$, *then*
  $$\int_{a_{1}}^{b_{1}} \int_{a_{2}}^{b_{2}} \int _{a_{3}}^{b_{3}}f(x_{1},x_{2},x_{3}) \diamond_{\alpha} x_{1}\diamond _{\alpha} x_{2} \diamond_{\alpha} x_{3}\geq0; $$
(ii) *If* $f(x_{1},x_{2},x_{3})\leq g(x_{1},x_{2},x_{3})$ *for all* $x_{i}\in[a_{i}, b_{i}]_{\mathbb{T}}$ $(i=1,2,3)$, *then*
  $$\begin{aligned} & \int_{a_{1}}^{b_{1}} \int_{a_{2}}^{b_{2}} \int _{a_{3}}^{b_{3}}f(x_{1},x_{2},x_{3}) \diamond_{\alpha} x_{1}\diamond _{\alpha} x_{2} \diamond_{\alpha} x_{3} \\ &\quad\leq \int_{a_{1}}^{b_{1}} \int_{a_{2}}^{b_{2}} \int _{a_{3}}^{b_{3}}g(x_{1},x_{2},x_{3}) \diamond_{\alpha} x_{1}\diamond _{\alpha} x_{2} \diamond_{\alpha} x_{3}; \end{aligned}$$
(iii) *If* $f(x_{1},x_{2},x_{3})\geq0$ *for all* $x_{i}\in[a_{i}, b_{i}]_{\mathbb{T}}$ $(i=1,2,3)$, *then* $f(x_{1},x_{2},x_{3})=0$ *if and only if*
  $$\int_{a_{1}}^{b_{1}} \int_{a_{2}}^{b_{2}} \int _{a_{3}}^{b_{3}}f(x_{1},x_{2},x_{3}) \diamond_{\alpha} x_{1}\diamond _{\alpha} x_{2} \diamond_{\alpha} x_{3}=0. $$

To prove the main results, we need the following lemmas.

### Lemma 2.2

(Bernoulli’s inequality; see [28])

*If* $x>0$ *and* $p>1$, *then*

8 $$ x^{p}\geq px+1-p. $$

### Lemma 2.3

(Young inequality; see [11])

*Let* $a,b>0$.

(i) *If* $p,q>0$ *with* $\frac{1}{p}+\frac{1}{q}=1$, *then*
  9 $$ ab \leq\frac{1}{p}a^{p}+\frac{1}{q}b^{q}. $$
(ii) *If* $p>0$, $q<0$ *with* $\frac{1}{p}+\frac{1}{q}=1$, *then*
  10 $$ ab \geq\frac{1}{p}a^{p}+\frac{1}{q}b^{q}. $$

### Lemma 2.4

(AG inequality; see [11])

*Let* $\alpha_{i}>0$ $(i=1,2,3)$.

(i) *If* $1<\lambda_{1}$, $\lambda_{2}, \ldots, \lambda_{n}<\infty$ *with* $\sum_{i=1}^{n}\frac{1}{\lambda_{i}}=1$, *then*
  11 $$ \prod_{i=1}^{n} \alpha_{i} \leq\sum_{i=1}^{n} \frac{\alpha_{i}^{\lambda_{i}}}{\lambda_{i}}. $$
(ii) *If* $\lambda_{1}>0$, $\lambda_{2}, \dots,\lambda_{m}<0$ *with* $\sum_{i=1}^{m}\frac{1}{\lambda_{i}}=1$, *then*
  12 $$ \prod_{i=1}^{m} \alpha_{i} \geq\sum_{i=1}^{m} \frac{\alpha_{i}^{\lambda_{i}}}{\lambda_{i}}. $$

### Lemma 2.5

(Schlömilch’s inequality for triple Diamond-Alpha integral)

*Suppose that* $\mathbb{T}$ *is a time scale*, $a_{i}, b_{i}\in\mathbb{T}$ *with* $a_{i}< b_{i}$ $(i=1,2,3)$, *and* $f(x_{1},x_{2},x_{3}), \omega (x_{1},x_{2},x_{3}):[a_{i},b_{i}]_{\mathbb{T}}^{3}\rightarrow[0,+\infty )$ *are* $\diamond_{\alpha}$-*integrable functions with* $\int _{a_{1}}^{b_{1}}\int_{a_{2}}^{b_{2}}\int_{a_{3}}^{b_{3}} \omega(x_{1},x_{2},x_{3})\diamond_{\alpha} x_{1}\diamond_{\alpha} x_{2}\diamond_{\alpha} x_{3}>0$. *Then*, *for* $s>r>0$, *we have*

$$\begin{aligned} & \biggl(\frac{\int_{a_{1}}^{b_{1}}\int_{a_{2}}^{b_{2}}\int_{a_{3}}^{b_{3}} \omega(x_{1},x_{2},x_{3} )f^{s}(x_{1},x_{2},x_{3}) \diamond_{\alpha} x_{1}\diamond_{\alpha} x_{2}\diamond_{\alpha} x_{3}}{ \int_{a_{1}}^{b_{1}}\int_{a_{2}}^{b_{2}}\int_{a_{3}}^{b_{3}} \omega(x_{1},x_{2},x_{3})\diamond_{\alpha} x_{1}\diamond_{\alpha} x_{2}\diamond_{\alpha} x_{3}} \biggr)^{\frac{1}{s}} \\ &\quad\geq \biggl(\frac{\int_{a_{1}}^{b_{1}}\int_{a_{2}}^{b_{2}}\int_{a_{3}}^{b_{3}} \omega(x_{1},x_{2},x_{3})f^{r}(x_{1},x_{2},x_{3}) \diamond_{\alpha} x_{1}\diamond_{\alpha} x_{2}\diamond_{\alpha} x_{3}}{ \int_{a_{1}}^{b_{1}}\int_{a_{2}}^{b_{2}}\int_{a_{3}}^{b_{3}} \omega(x_{1},x_{2},x_{3})\diamond_{\alpha} x_{1}\diamond_{\alpha} x_{2}\diamond_{\alpha} x_{3}} \biggr)^{\frac{1}{r}}. \end{aligned}$$

### Proof

Without loss of generality, we may suppose that $\int _{a_{1}}^{b_{1}}\int_{a_{2}}^{b_{2}}\int _{a_{3}}^{b_{3}}h(x_{1},x_{2},x_{3})\diamond_{\alpha} x_{1}\diamond _{\alpha} x_{2}\diamond_{\alpha} x_{3}=1$. If $s>r>0$, then $\frac{s}{r}>1$. Therefore, by inequality (8) we have

$$\begin{aligned} & \int_{a_{1}}^{b_{1}} \int_{a_{2}}^{b_{2}} \int_{a_{3}}^{b_{3}} \biggl[\biggl( \frac{f(x_{1},x_{2},x_{3})}{ \int_{a_{1}}^{b_{1}}\int_{a_{2}}^{b_{2}}\int_{a_{3}}^{b_{3}} \omega(x_{1},x_{2},x_{3})f(x_{1},x_{2},x_{3})\diamond_{\alpha} x_{1}\diamond_{\alpha} x_{2}\diamond_{\alpha} x_{3}} \biggr)^{\frac{s}{r}} \\ &\qquad{} \times \omega(x_{1},x_{2},x_{3})\biggr] \diamond_{\alpha} x_{1}\diamond_{\alpha} x_{2} \diamond_{\alpha} x_{3} \\ &\quad\geq \int_{a_{1}}^{b_{1}} \int_{a_{2}}^{b_{2}} \int_{a_{3}}^{b_{3}} \biggl[\biggl( \frac{\frac{s}{r}f(x_{1},x_{2},x_{3})}{\int_{a}^{b}\int_{a}^{b} \cdots\int_{a}^{b}\omega(x_{1},x_{2},x_{3})f(x_{1},x_{2},x_{3})\diamond _{\alpha} x_{1}\diamond_{\alpha} x_{2}\diamond_{\alpha} x_{3}}+1- \frac {s}{r} \biggr) \\ &\qquad{} \times \omega(x_{1},x_{2},x_{3})\biggr] \diamond_{\alpha} x_{1}\diamond_{\alpha} x_{2} \diamond_{\alpha} x_{3} \\ &\quad= 1, \end{aligned}$$

which implies

13 $$\begin{aligned} & \int_{a_{1}}^{b_{1}} \int_{a_{2}}^{b_{2}} \int_{a_{3}}^{b_{3}} \omega(x_{1},x_{2},x_{3} )f^{\frac{s}{r}}(x_{1},x_{2},x_{3})\diamond _{\alpha} x_{1}\diamond_{\alpha} x_{2} \diamond_{\alpha} x_{3} \\ &\quad \geq \biggl( \int_{a_{1}}^{b_{1}} \int_{a_{2}}^{b_{2}} \int_{a_{3}}^{b_{3}} \omega(x_{1},x_{2},x_{3})f(x_{1},x_{2},x_{3}) \diamond_{\alpha} x_{1}\diamond_{\alpha} x_{2} \diamond_{\alpha} x_{3} \biggr)^{\frac{s}{r}}. \end{aligned}$$

Replacing *f* by $f^{r}$ in (13), we find

$$\begin{aligned} & \int_{a_{1}}^{b_{1}} \int_{a_{2}}^{b_{2}} \int_{a_{3}}^{b_{3}} \omega(x_{1},x_{2},x_{3} )f^{s}(x_{1},x_{2},x_{3}) \diamond_{\alpha} x_{1}\diamond_{\alpha} x_{2} \diamond_{\alpha} x_{3} \\ &\quad \geq \biggl( \int_{a_{1}}^{b_{1}} \int_{a_{2}}^{b_{2}} \int_{a_{3}}^{b_{3}} \omega(x_{1},x_{2},x_{3} )f^{r}(x_{1},x_{2},x_{3}) \diamond_{\alpha} x_{1}\diamond_{\alpha} x_{2} \diamond_{\alpha} x_{3} \biggr)^{\frac{s}{r}}. \end{aligned}$$

Then

$$\begin{aligned} & \biggl( \int_{a_{1}}^{b_{1}} \int_{a_{2}}^{b_{2}} \int_{a_{3}}^{b_{3}} \omega(x_{1},x_{2},x_{3} )f^{s}(x_{1},x_{2},x_{3}) \diamond_{\alpha} x_{1}\diamond_{\alpha} x_{2} \diamond_{\alpha} x_{3} \biggr)^{\frac{1}{s}} \\ &\quad \geq \biggl( \int_{a_{1}}^{b_{1}} \int_{a_{2}}^{b_{2}} \int_{a_{3}}^{b_{3}} \omega(x_{1},x_{2},x_{3} )f^{r}(x_{1},x_{2},x_{3}) \diamond_{\alpha} x_{1}\diamond_{\alpha} x_{2} \diamond_{\alpha} x_{3} \biggr)^{\frac{1}{r}}. \end{aligned}$$

Thus, the proof of Lemma 2.5 is completed. □

Now, we give the following Hölder and reverse Hölder inequalities for triple Diamond-Alpha integral.

### Theorem 2.6

*Suppose that* $\mathbb{T}$ *is a time scale*, $a_{i},b_{i}\in\mathbb{T}$ *with* $a_{i}< b_{i}$ $(i=1,2,3)$, *and* $f(x_{1},x_{2},x_{3})$, $g(x_{1},x_{2},x_{3})$, $\omega(x_{1},x_{2},x_{3}): [a_{i},b_{i}]_{\mathbb{T}}^{3}\rightarrow \mathbb{R}$ *are* $\diamond_{\alpha}$-*integrable functions*.

(i) *If* $p, q>0$ *are such that* $\frac{1}{p}+\frac{1}{q}=1$, *then*
  14 $$\begin{aligned} & \int_{a_{1}}^{b_{1}} \int_{a_{2}}^{b_{2}} \int_{a_{3}}^{b_{3}} \bigl\vert \omega (x_{1},x_{2},x_{3})f(x_{1},x_{2},x_{3})g(x_{1},x_{2},x_{3}) \bigr\vert \diamond_{\alpha} x_{1}\diamond_{\alpha} x_{2}\diamond_{\alpha} x_{3} \\ &\quad\leq \biggl( \int_{a_{1}}^{b_{1}} \int_{a_{2}}^{b_{2}} \int_{a_{3}}^{b_{3}} \bigl\vert \omega(x_{1},x_{2},x_{3} ) \bigr\vert \bigl\vert f(x_{1},x_{2},x_{3}) \bigr\vert ^{p}\diamond_{\alpha} x_{1}\diamond _{\alpha} x_{2}\diamond_{\alpha} x_{3} \biggr)^{\frac{1}{p}} \\ & \qquad{}\times \biggl( \int_{a_{1}}^{b_{1}} \int_{a_{2}}^{b_{2}} \int_{a_{3}}^{b_{3}} \bigl\vert \omega(x_{1},x_{2},x_{3} ) \bigr\vert \bigl\vert g(x_{1},x_{2},x_{3}) \bigr\vert ^{q} \diamond_{\alpha} x_{1}\diamond _{\alpha} x_{2}\diamond_{\alpha} x_{3} \biggr)^{\frac{1}{q}} . \end{aligned}$$
(ii) *If* $q<0$ *is such that* $\frac{1}{p}+\frac{1}{q}=1$, *then*
  15 $$\begin{aligned} & \int_{a_{1}}^{b_{1}} \int_{a_{2}}^{b_{2}} \int_{a_{3}}^{b_{3}} \bigl\vert \omega (x_{1},x_{2},x_{3})f(x_{1},x_{2},x_{3})g(x_{1},x_{2},x_{3}) \bigr\vert \diamond_{\alpha} x_{1}\diamond_{\alpha} x_{2}\diamond_{\alpha} x_{3} \\ &\quad\geq \biggl( \int_{a_{1}}^{b_{1}} \int_{a_{2}}^{b_{2}} \int_{a_{3}}^{b_{3}} \bigl\vert \omega(x_{1},x_{2},x_{3} ) \bigr\vert \bigl\vert f(x_{1},x_{2},x_{3}) \bigr\vert ^{p}\diamond_{\alpha} x_{1}\diamond _{\alpha} x_{2}\diamond_{\alpha} x_{3} \biggr)^{\frac{1}{p}} \\ & \qquad{}\times \biggl( \int_{a_{1}}^{b_{1}} \int_{a_{2}}^{b_{2}} \int_{a_{3}}^{b_{3}} \bigl\vert \omega(x_{1},x_{2},x_{3} ) \bigr\vert \bigl\vert g(x_{1},x_{2},x_{3}) \bigr\vert ^{q} \diamond_{\alpha} x_{1}\diamond _{\alpha} x_{2}\diamond_{\alpha} x_{3} \biggr)^{\frac{1}{q}} . \end{aligned}$$

### Proof

Case (i): Let $p,q>0$ with $\frac{1}{p}+\frac{1}{q}=1$. Without loss of generality, we may suppose that

$$\int_{a_{1}}^{b_{1}} \int_{a_{2}}^{b_{2}} \int_{a_{3}}^{b_{3}} \bigl\vert \omega(x_{1},x_{2},x_{3} ) \bigr\vert \bigl\vert f(x_{1},x_{2},x_{3}) \bigr\vert ^{p}\diamond_{\alpha} x_{1} \diamond_{\alpha} x_{2}\diamond _{\alpha} x_{3} \neq0 $$

and

$$\int_{a_{1}}^{b_{1}} \int_{a_{2}}^{b_{2}} \int_{a_{3}}^{b_{3}} \bigl\vert \omega(x_{1},x_{2},x_{3} ) \bigr\vert \bigl\vert g(x_{1},x_{2},x_{3}) \bigr\vert ^{q}\diamond_{\alpha} x_{1} \diamond_{\alpha} x_{2}\diamond _{\alpha} x_{3} \neq0. $$

Let

$$\mu =\frac{ \vert \omega(x_{1},x_{2},x_{3}) \vert ^{\frac {1}{p}} \vert f(x_{1},x_{2},x_{3}) \vert }{ (\int_{a_{1}}^{b_{1}}\int_{a_{2}}^{b_{2}}\int_{a_{3}}^{b_{3}} \vert \omega(x_{1},x_{2},x_{3} ) \vert \vert f(x_{1},x_{2},x_{3}) \vert ^{p}\diamond_{\alpha} x_{1}\diamond_{\alpha} x_{2}\diamond _{\alpha} x_{3} )^{\frac{1}{p}}} $$

and

$$\nu =\frac{ \vert \omega(x_{1},x_{2},x_{3}) \vert ^{\frac {1}{q}} \vert g(x_{1},x_{2},x_{3}) \vert }{ (\int_{a_{1}}^{b_{1}}\int_{a_{2}}^{b_{2}}\int_{a_{3}}^{b_{3}} \vert \omega(x_{1},x_{2},x_{3} ) \vert \vert g(x_{1},x_{2},x_{3}) \vert ^{q}\diamond_{\alpha} x_{1}\diamond_{\alpha} x_{2}\diamond _{\alpha} x_{3} )^{\frac{1}{q}}}. $$

From the Young inequality (9) we get

$$\begin{aligned} & \int_{a_{1}}^{b_{1}} \int_{a_{2}}^{b_{2}} \int_{a_{3}}^{b_{3}}\mu\nu \diamond_{\alpha} x_{1}\diamond_{\alpha} x_{2}\diamond_{\alpha} x_{3} \\ &\quad\leq \int_{a_{1}}^{b_{1}} \int_{a_{2}}^{b_{2}} \int_{a_{3}}^{b_{3}} \biggl(\frac{\mu^{p}}{p}+ \frac{\nu^{q}}{q} \biggr) \diamond_{\alpha} x_{1} \diamond_{\alpha} x_{2}\diamond_{\alpha} x_{3} \\ &\quad=\frac{1}{p} \int_{a_{1}}^{b_{1}} \int_{a_{2}}^{b_{2}} \int_{a_{3}}^{b_{3}} \biggl(\frac{ \vert \omega(x_{1},x_{2},x_{3}) \vert }{ \int_{a_{1}}^{b_{1}}\int_{a_{2}}^{b_{2}}\int_{a_{3}}^{b_{3}} \vert \omega(x_{1},x_{2},x_{3} ) \vert \vert f(x_{1},x_{2},x_{3}) \vert ^{p}\diamond_{\alpha} x_{1}\diamond _{\alpha} x_{2}\diamond_{\alpha} x_{3}} \\ &\qquad{}\times \bigl\vert f(x_{1},x_{2},x_{3}) \bigr\vert ^{p}\biggr)\diamond_{\alpha} x_{1}\diamond_{\alpha} x_{2} \diamond_{\alpha} x_{3} \\ &\qquad{} +\frac{1}{q} \int_{a_{1}}^{b_{1}} \int_{a_{2}}^{b_{2}} \int_{a_{3}}^{b_{3}} \biggl(\frac{ \vert \omega(x_{1},x_{2},x_{3}) \vert }{ \int_{a_{1}}^{b_{1}}\int_{a_{2}}^{b_{2}}\int_{a_{3}}^{b_{3}} \vert \omega(x_{1},x_{2},x_{3} ) \vert \vert f(x_{1},x_{2},x_{3}) \vert ^{q}\diamond_{\alpha} x_{1}\diamond _{\alpha} x_{2}\diamond_{\alpha} x_{3}} \\ &\qquad{}\times \bigl\vert f(x_{1},x_{2},x_{3}) \bigr\vert ^{q}\biggr)\diamond_{\alpha} x_{1}\diamond_{\alpha} x_{2} \diamond_{\alpha} x_{3} \\ &\quad=\frac{1}{p}+\frac{1}{q}=1. \end{aligned}$$

Therefore, we get the desired inequality (14).

Case (ii). Let $q<0$ with $\frac{1}{p}+\frac{1}{q}=1$. Write $\alpha =-\frac{p}{q}, \beta=\frac{1}{q}$. Then $\frac{1}{\alpha}+\frac{1}{\beta }=1$ with $\alpha, \beta>0$. Setting $f(x_{1},x_{2},x_{3})=F(x_{1},x_{2},x_{3})$ and $g(x_{1},x_{2},x_{3})=G(x_{1},x_{2},x_{3})$ in (14), we get

16 $$\begin{aligned} & \int_{a_{1}}^{b_{1}} \int_{a_{2}}^{b_{2}} \int_{a_{3}}^{b_{3}} \bigl\vert \omega(x_{1},x_{2},x_{3}) F(x_{1},x_{2},x_{3})G(x_{1},x_{2},x_{3}) \bigr\vert \diamond_{\alpha} x_{1}\diamond_{\alpha} x_{2}\diamond_{\alpha} x_{3} \\ &\quad\leq \biggl( \int_{a_{1}}^{b_{1}} \int_{a_{2}}^{b_{2}} \int_{a_{3}}^{b_{3}} \bigl\vert \omega(x_{1},x_{2},x_{3} ) \bigr\vert \bigl\vert F(x_{1},x_{2},x_{3}) \bigr\vert ^{\alpha}\diamond_{\alpha} x_{1}\diamond _{\alpha} x_{2}\diamond_{\alpha} x_{3} \biggr)^{\frac{1}{\alpha}} \\ &\qquad{} \times \biggl( \int_{a_{1}}^{b_{1}} \int_{a_{2}}^{b_{2}} \int_{a_{3}}^{b_{3}} \bigl\vert \omega(x_{1},x_{2},x_{3}) \bigr\vert \bigl\vert G(x_{1},x_{2},x_{3}) \bigr\vert ^{\beta} \diamond_{\alpha} x_{1}\diamond _{\alpha} x_{2}\diamond_{\alpha} x_{3} \biggr)^{\frac{1}{\beta}} . \end{aligned}$$

Putting $F(x_{1},x_{2},x_{3})=f^{-q}(x_{1},x_{2},x_{3})$ and $G(x_{1},x_{2},x_{3})=f^{q}(x_{1},x_{2},x_{3})g^{q}(x_{1},x_{2},x_{3})$ in equality (16), we immediately obtain the desired inequality (15). □

Next, we present the following generalizations of inequalities (14) and (15).

### Theorem 2.7

*Suppose that* $\mathbb{T}$ *is a time scale*, $a_{i},b_{i}\in\mathbb{T}$ *with* $a_{i}< b_{i}$ $(i=1,2,3)$, *and* $f(x_{1},x_{2},x_{3})$, $g(x_{1},x_{2},x_{3})$, $\omega(x_{1},x_{2},x_{3}): [a_{i},b_{i}]_{\mathbb{T}}^{3}\rightarrow \mathbb{R}$ *are* $\diamond_{\alpha}$-*integrable functions*.

(i) *Let* $\frac{1}{p}+\frac{1}{q}=\frac{1}{r}$ *with* $p,q,r\in\mathbb {R}-\{0\}$, $p>0$ *and* $q>0$ *or* $p>0$, $q<0$, *and* $r<0$. *Then*
  17 $$\begin{aligned} & \biggl( \int_{a_{1}}^{b_{1}} \int_{a_{2}}^{b_{2}} \int_{a_{3}}^{b_{3}} \bigl\vert \omega (x_{1},x_{2},x_{3})f^{r}(x_{1},x_{2},x_{3})g^{r}(x_{1},x_{2},x_{3}) \bigr\vert \diamond_{\alpha} x_{1}\diamond_{\alpha} x_{2}\diamond_{\alpha} x_{3} \biggr)^{\frac{1}{r}} \\ &\quad\leq \biggl( \int_{a_{1}}^{b_{1}} \int_{a_{2}}^{b_{2}} \int_{a_{3}}^{b_{3}} \bigl\vert \omega(x_{1},x_{2},x_{3}) \bigr\vert \bigl\vert f(x_{1},x_{2},x_{3}) \bigr\vert ^{p}\diamond_{\alpha} x_{1}\diamond _{\alpha} x_{2}\diamond_{\alpha} x_{3} \biggr)^{\frac{1}{p}} \\ &\qquad{} \times \biggl( \int_{a_{1}}^{b_{1}} \int_{a_{2}}^{b_{2}} \int_{a_{3}}^{b_{3}} \bigl\vert \omega(x_{1},x_{2},x_{3}) \bigr\vert \bigl\vert g(x_{1},x_{2},x_{3}) \bigr\vert ^{q} \diamond_{\alpha} x_{1}\diamond _{\alpha} x_{2}\diamond_{\alpha} x_{3} \biggr)^{\frac{1}{q}} . \end{aligned}$$
(ii) *Let* $\frac{1}{p}+\frac{1}{q}=\frac{1}{r}$ *with* $p,q,r\in\mathbb {R}-\{0\}$, $p>0$, $q<0$, *and* $r>0$ *or* $p<0$ *and* $q<0$. *Then*
  18 $$\begin{aligned} & \biggl( \int_{a_{1}}^{b_{1}} \int_{a_{2}}^{b_{2}} \int_{a_{3}}^{b_{3}} \bigl\vert \omega (x_{1},x_{2},x_{3})f^{r}(x_{1},x_{2},x_{3})g^{r}(x_{1},x_{2},x_{3}) \bigr\vert \diamond_{\alpha} x_{1}\diamond_{\alpha} x_{2}\diamond_{\alpha} x_{3} \biggr)^{\frac{1}{r}} \\ &\quad\geq \biggl( \int_{a_{1}}^{b_{1}} \int_{a_{2}}^{b_{2}} \int_{a_{3}}^{b_{3}} \bigl\vert \omega(x_{1},x_{2},x_{3}) \bigr\vert \bigl\vert f(x_{1},x_{2},x_{3}) \bigr\vert ^{p}\diamond_{\alpha} x_{1}\diamond _{\alpha} x_{2}\diamond_{\alpha} x_{3} \biggr)^{\frac{1}{p}} \\ &\qquad{} \times \biggl( \int_{a_{1}}^{b_{1}} \int_{a_{2}}^{b_{2}} \int_{a_{3}}^{b_{3}} \bigl\vert \omega(x_{1},x_{2},x_{3}) \bigr\vert \bigl\vert g(x_{1},x_{2},x_{3}) \bigr\vert ^{q} \diamond_{\alpha} x_{1}\diamond _{\alpha} x_{2}\diamond_{\alpha} x_{3} \biggr)^{\frac{1}{q}} . \end{aligned}$$

### Proof

(i) Case 1. When $p>0$, $q>0$, by $\frac{1}{p}+\frac{1}{q}=\frac {1}{r}$ we get

$$\frac{p}{r}>1, \qquad\frac{1}{p/r}+\frac{1}{q/r}=1. $$

Then, by (14) we find that

$$\begin{aligned} & \int_{a_{1}}^{b_{1}} \int_{a_{2}}^{b_{2}} \int_{a_{3}}^{b_{3}} \bigl\vert \omega (x_{1},x_{2},x_{3})f^{r}(x_{1},x_{2},x_{3})g^{r}(x_{1},x_{2},x_{3}) \bigr\vert \diamond_{\alpha} x_{1}\diamond_{\alpha} x_{2}\diamond_{\alpha} x_{3} \\ &\quad\leq \biggl( \int_{a_{1}}^{b_{1}} \int_{a_{2}}^{b_{2}} \int_{a_{3}}^{b_{3}} \bigl\vert \omega(x_{1},x_{2},x_{3})f^{r}(x_{1},x_{2},x_{3}) \bigr\vert ^{\frac{p}{r}}\diamond_{\alpha} x_{1} \diamond_{\alpha} x_{2}\diamond_{\alpha} x_{3} \biggr)^{\frac{r}{p}} \\ &\qquad{} \times \biggl( \int_{a_{1}}^{b_{1}} \int_{a_{2}}^{b_{2}} \int_{a_{3}}^{b_{3}} \bigl\vert \omega(x_{1},x_{2},x_{3})g^{r}(x_{1},x_{2},x_{3}) \bigr\vert ^{\frac{q}{r}}\diamond_{\alpha} x_{1} \diamond_{\alpha} x_{2}\diamond_{\alpha} x_{3} \biggr)^{\frac{r}{q}}. \end{aligned}$$

Therefore

$$\begin{aligned} &\biggl( \int_{a_{1}}^{b_{1}} \int_{a_{2}}^{b_{2}} \int_{a_{3}}^{b_{3}} \bigl\vert \omega (x_{1},x_{2},x_{3})f^{r}(x_{1},x_{2},x_{3})g^{r}(x_{1},x_{2},x_{3}) \bigr\vert \diamond_{\alpha} x_{1}\diamond_{\alpha} x_{2}\diamond_{\alpha} x_{3} \biggr)^{\frac{1}{r}} \\ &\quad\leq \biggl( \int_{a_{1}}^{b_{1}} \int_{a_{2}}^{b_{2}} \int_{a_{3}}^{b_{3}} \bigl\vert \omega(x_{1},x_{2},x_{3})f^{r}(x_{1},x_{2},x_{3}) \bigr\vert ^{\frac{p}{r}}\diamond_{\alpha} x_{1} \diamond_{\alpha} x_{2}\diamond_{\alpha} x_{3} \biggr)^{\frac{1}{p}} \\ & \qquad{}\times \biggl( \int_{a_{1}}^{b_{1}} \int_{a_{2}}^{b_{2}} \int_{a_{3}}^{b_{3}} \bigl\vert \omega(x_{1},x_{2},x_{3})g^{r}(x_{1},x_{2},x_{3}) \bigr\vert ^{\frac{q}{r}}\diamond_{\alpha} x_{1} \diamond_{\alpha} x_{2}\diamond_{\alpha} x_{3} \biggr)^{\frac{1}{q}}. \end{aligned}$$

Case 2. When $p>0$, $q<0$, and $r<0$, by the same method as in Case 1, we can obtain inequality (17).

(ii) Case I. When $p>0$, $q<0$, and $r>0$, we find from $\frac {1}{p}+\frac{1}{q}=\frac{1}{r}$ that

$$\frac{r}{p}>1, \qquad \frac{1}{r/p}+\frac{1}{-q/p} = \frac{p}{r}- \frac{p}{q}=p \biggl(\frac{1}{r}- \frac{1}{q} \biggr)=1. $$

Then, by inequality (14) we get

$$\begin{aligned} & \int_{a_{1}}^{b_{1}} \int_{a_{2}}^{b_{2}} \int_{a_{3}}^{b_{3}} \bigl\vert \omega(x_{1},x_{2},x_{3})f^{p}(x_{1},x_{2},x_{3}) \bigr\vert \diamond_{\alpha} x_{1}\diamond_{\alpha} x_{2}\diamond_{\alpha} x_{3} \\ &\quad= \int_{a_{1}}^{b_{1}} \int_{a_{2}}^{b_{2}} \int_{a_{3}}^{b_{3}} \biggl( \bigl\vert \omega(x_{1},x_{2},x_{3})f^{p}(x_{1},x_{2},x_{3})\bigr\vert \\ &\qquad{}\times\bigl\vert g^{p}(x_{1},x_{2},x_{3})g^{-p}(x_{1},x_{2},x_{3}) \bigr\vert \biggr) \diamond_{\alpha} x_{1}\diamond_{\alpha} x_{2}\diamond _{\alpha} x_{3} \\ &\quad\leq \biggl( \int_{a_{1}}^{b_{1}} \int_{a_{2}}^{b_{2}} \int_{a_{3}}^{b_{3}} \bigl\vert \omega(x_{1},x_{2},x_{3}) \bigr\vert \bigl\vert f^{p}(x_{1},x_{2},x_{3})g^{p}(x_{1},x_{2},x_{3}) \bigr\vert ^{\frac{r}{p}}\diamond_{\alpha} x_{1} \diamond_{\alpha} x_{2}\diamond_{\alpha} x_{3} \biggr)^{\frac{p}{r}} \\ &\qquad{} \times \biggl( \int_{a_{1}}^{b_{1}} \int_{a_{2}}^{b_{2}} \int_{a_{3}}^{b_{3}} \bigl\vert \omega(x_{1},x_{2},x_{3}) \bigr\vert \bigl\vert g^{-p}(x_{1},x_{2},x_{3}) \bigr\vert ^{-\frac{q}{p}}\diamond_{\alpha} x_{1} \diamond_{\alpha} x_{2}\diamond_{\alpha} x_{3} \biggr)^{-\frac{p}{q}} \\ &\quad= \biggl( \int_{a_{1}}^{b_{1}} \int_{a_{2}}^{b_{2}} \int_{a_{3}}^{b_{3}} \bigl\vert \omega(x_{1},x_{2},x_{3}) \bigr\vert \bigl\vert f^{r}(x_{1},x_{2},x_{3})g^{r}(x_{1},x_{2},x_{3}) \bigr\vert \diamond_{\alpha} x_{1}\diamond_{\alpha} x_{2}\diamond _{\alpha} x_{3} \biggr)^{\frac{p}{r}} \\ & \qquad{}\times \biggl( \int_{a_{1}}^{b_{1}} \int_{a_{2}}^{b_{2}} \int_{a_{3}}^{b_{3}} \bigl\vert \omega(x_{1},x_{2},x_{3}) \bigr\vert \bigl\vert g^{q}(x_{1},x_{2},x_{3}) \bigr\vert \diamond_{\alpha} x_{1}\diamond_{\alpha} x_{2}\diamond _{\alpha} x_{3} \biggr)^{-\frac{p}{q}}, \end{aligned}$$

which implies

19 $$\begin{aligned} & \biggl( \int_{a_{1}}^{b_{1}} \int_{a_{2}}^{b_{2}} \int_{a_{3}}^{b_{3}} \bigl\vert \omega(x_{1},x_{2},x_{3})f^{p}(x_{1},x_{2},x_{3}) \bigr\vert \diamond_{\alpha} x_{1}\diamond_{\alpha} x_{2}\diamond_{\alpha} x_{3} \biggr)^{\frac{1}{p}} \\ &\quad\leq \biggl( \int_{a_{1}}^{b_{1}} \int_{a_{2}}^{b_{2}} \int_{a_{3}}^{b_{3}} \bigl\vert \omega(x_{1},x_{2},x_{3}) \bigr\vert \bigl\vert f^{r}(x_{1},x_{2},x_{3})g^{r}(x_{1},x_{2},x_{3}) \bigr\vert \diamond_{\alpha} x_{1}\diamond_{\alpha} x_{2}\diamond _{\alpha} x_{3} \biggr)^{\frac{1}{r}} \\ &\qquad{} \times \biggl( \int_{a_{1}}^{b_{1}} \int_{a_{2}}^{b_{2}} \int_{a_{3}}^{b_{3}} \bigl\vert \omega(x_{1},x_{2},x_{3}) \bigr\vert \bigl\vert g^{q}(x_{1},x_{2},x_{3}) \bigr\vert \diamond_{\alpha} x_{1}\diamond_{\alpha} x_{2}\diamond _{\alpha} x_{3} \biggr)^{-\frac{1}{q}}. \end{aligned}$$

Thus from inequality (19) we obtain

$$\begin{aligned} & \biggl( \int_{a_{1}}^{b_{1}} \int_{a_{2}}^{b_{2}} \int_{a_{3}}^{b_{3}} \bigl\vert \omega (x_{1},x_{2},x_{3})f^{r}(x_{1},x_{2},x_{3})g^{r}(x_{1},x_{2},x_{3}) \bigr\vert \diamond_{\alpha} x_{1}\diamond_{\alpha} x_{2}\diamond_{\alpha} x_{3} \biggr)^{\frac{1}{r}} \\ &\quad\geq \biggl( \int_{a_{1}}^{b_{1}} \int_{a_{2}}^{b_{2}} \int_{a_{3}}^{b_{3}} \bigl\vert \omega(x_{1},x_{2},x_{3}) \bigr\vert \bigl\vert f(x_{1},x_{2},x_{3}) \bigr\vert ^{p}\diamond_{\alpha} x_{1}\diamond _{\alpha} x_{2}\diamond_{\alpha} x_{3} \biggr)^{\frac{1}{p}} \\ &\qquad{} \times \biggl( \int_{a_{1}}^{b_{1}} \int_{a_{2}}^{b_{2}} \int_{a_{3}}^{b_{3}} \bigl\vert \omega(x_{1},x_{2},x_{3}) \bigr\vert \bigl\vert g(x_{1},x_{2},x_{3}) \bigr\vert ^{q} \diamond_{\alpha} x_{1}\diamond _{\alpha} x_{2}\diamond_{\alpha} x_{3} \biggr)^{\frac{1}{q}} . \end{aligned}$$

Case II. When $p<0$ and $q<0$, by the same method as in Case I, we can obtain the desired inequality (18). The proof of Theorem 2.7 is completed. □

We present another generalization of inequality (14).

### Theorem 2.8

*Suppose that* $\mathbb{T}$ *is a time scale*, $a_{i},b_{i}\in\mathbb{T}$ *with* $a_{i}< b_{i}$ $(i=1,2,3)$, *and that* $f(x_{1},x_{2},x_{3})$, $g(x_{1},x_{2},x_{3})$, $\omega(x_{1},x_{2},x_{3}): [a_{i},b_{i}]_{\mathbb{T}}^{3}\rightarrow \mathbb{R}$ *are* $\diamond_{\alpha}$-*integrable functions*. *If* $p>0$, $q>0$ *are such that* $0<\frac{1}{p}+\frac{1}{q}<1$, *then*

20 $$\begin{aligned} & \int_{a_{1}}^{b_{1}} \int_{a_{2}}^{b_{2}} \int_{a_{3}}^{b_{3}} \bigl\vert \omega(x_{1},x_{2},x_{3}) f(x_{1},x_{2},x_{3})g(x_{1},x_{2},x_{3}) \bigr\vert \diamond_{\alpha} x_{1}\diamond_{\alpha} x_{2}\diamond_{\alpha} x_{3} \\ &\quad\leq \biggl( \int_{a_{1}}^{b_{1}} \int_{a_{2}}^{b_{2}} \int_{a_{3}}^{b_{3}} \bigl\vert \omega(x_{1},x_{2},x_{3}) \bigr\vert \bigl\vert f(x_{1},x_{2},x_{3}) \bigr\vert ^{p}\diamond_{\alpha} x_{1}\diamond _{\alpha} x_{2}\diamond_{\alpha} x_{3} \biggr)^{\frac{1}{p}} \\ &\qquad{}\times \biggl( \int_{a_{1}}^{b_{1}} \int_{a_{2}}^{b_{2}} \int_{a_{3}}^{b_{3}} \bigl\vert \omega(x_{1},x_{2},x_{3}) \bigr\vert \bigl\vert g(x_{1},x_{2},x_{3}) \bigr\vert ^{q} \diamond_{\alpha} x_{1}\diamond _{\alpha} x_{2}\diamond_{\alpha}x_{3} \biggr)^{\frac{1}{q}} . \end{aligned}$$

### Proof

Denote $\gamma:=\frac{1}{p}+\frac{1}{q}$, $\zeta=\gamma p$, $\delta =q\gamma$. Then $\zeta>1$, $\delta>1$, and $\frac{1}{\zeta}+\frac {1}{\delta}=1$. Hence from Lemma 2.5 with $\zeta< p$, $\delta< q$ and inequality (14) we find that

$$\begin{aligned} & \int_{a_{1}}^{b_{1}} \int_{a_{2}}^{b_{2}} \int_{a_{3}}^{b_{3}} \bigl\vert \omega (x_{1},x_{2},x_{3})f(x_{1},x_{2},x_{3})g(x_{1},x_{2},x_{3}) \bigr\vert \diamond_{\alpha} x_{1}\diamond_{\alpha} x_{2}\diamond_{\alpha} x_{3} \\ &\quad\leq \biggl( \int_{a_{1}}^{b_{1}} \int_{a_{2}}^{b_{2}} \int _{a_{3}}^{b_{3}} \bigl\vert \omega(x_{1},x_{2},x_{3}) \bigr\vert \bigl\vert f(x_{1},x_{2},x_{3}) \bigr\vert ^{\zeta}\diamond_{\alpha} x_{1} \diamond_{\alpha} x_{2}\diamond_{\alpha} x_{3} \biggr)^{\frac {1}{\zeta}} \\ &\qquad{} \times \biggl( \int_{a_{1}}^{b_{1}} \int_{a_{2}}^{b_{2}} \int_{a_{3}}^{b_{3}} \bigl\vert \omega(x_{1},x_{2},x_{3}) \bigr\vert \bigl\vert g(x_{1},x_{2},x_{3}) \bigr\vert ^{\delta} \diamond_{\alpha} x_{1} \diamond_{\alpha} x_{2}\diamond_{\alpha} x_{3} \biggr)^{\frac {1}{\delta}} \\ &\quad\leq \biggl( \int_{a_{1}}^{b_{1}} \int_{a_{2}}^{b_{2}} \int_{a_{3}}^{b_{3}} \bigl\vert \omega(x_{1},x_{2},x_{3}) \bigr\vert \bigl\vert f(x_{1},x_{2},x_{3}) \bigr\vert ^{p}\diamond_{\alpha} x_{1} \diamond_{\alpha} x_{2}\diamond _{\alpha} x_{3} \biggr)^{\frac{1}{p}} \\ &\qquad{} \times \biggl( \int_{a_{1}}^{b_{1}} \int_{a_{2}}^{b_{2}} \int_{a_{3}}^{b_{3}} \bigl\vert \omega(x_{1},x_{2},x_{3}) \bigr\vert \bigl\vert g(x_{1},x_{2},x_{3}) \bigr\vert ^{q} \diamond_{\alpha} x_{1}\diamond _{\alpha} x_{2}\cdots\diamond_{\alpha}x_{n} \biggr)^{\frac{1}{q}}. \end{aligned}$$

The proof of Theorem 2.8 is completed. □

### Theorem 2.9

*Suppose that* $\mathbb{T}$ *is a time scale*, $a_{i},b_{i}\in\mathbb{T}$ *with* $a_{i}< b_{i}$ $(i=1,2,3)$, *and that* $f_{i}(x_{1},x_{2},x_{3})\ (i=1,2,\ldots,m)$, $\omega(x_{1},x_{2},x_{3}): [a_{i},b_{i}]_{\mathbb{T}}^{3}\rightarrow \mathbb{R}$ *are* $\diamond_{\alpha}$-*integrable functions*.

(I) *Let* $1<\lambda_{1}$, $\lambda_{2}, \ldots, \lambda_{m}<\infty$ *such that* $\sum_{i=1}^{m}\frac{1}{\lambda_{i}}=1$. *Then*
  21 $$\begin{aligned} & \int_{a_{1}}^{b_{1}} \int_{a_{2}}^{b_{2}} \int_{a_{3}}^{b_{3}} \Biggl\vert \omega(x_{1},x_{2},x_{3}) \Biggl(\prod_{i=1}^{m}f_{i}(x_{1},x_{2},x_{3}) \Biggr) \Biggr\vert \diamond_{\alpha} x_{1} \diamond_{\alpha} x_{2}\diamond _{\alpha} x_{3} \\ &\quad\leq\prod_{i=1}^{m} \biggl( \int_{a_{1}}^{b_{1}} \int_{a_{2}}^{b_{2}} \int _{a_{3}}^{b_{3}} \bigl\vert \omega(x_{1},x_{2},x_{3}) \bigr\vert \bigl\vert f_{i}(x_{1},x_{2},x_{3}) \bigr\vert ^{\lambda_{i}}\diamond_{\alpha} x_{1} \diamond_{\alpha} x_{2}\diamond_{\alpha} x_{3} \biggr)^{\frac{1}{\lambda_{i}}}. \end{aligned}$$
(II) *Let* $\lambda_{1}>0$, $\lambda_{2}, \ldots, \lambda_{m}<0$ *be such that* $\sum_{i=1}^{m}\frac{1}{\lambda_{i}}=1$. *Then inequality* (21) *is reversed*.

### Proof

Case (I). Without loss of generality, we may suppose that

$$\prod_{i=1}^{m} \biggl( \int_{a_{1}}^{b_{1}} \int_{a_{2}}^{b_{2}} \int _{a_{3}}^{b_{3}} \bigl\vert \omega(x_{1},x_{2},x_{3}) \bigr\vert \bigl\vert f_{i}(x_{1},x_{2},x_{3}) \bigr\vert ^{\lambda_{i}}\diamond_{\alpha} x_{1} \diamond_{\alpha} x_{2}\diamond_{\alpha} x_{3} \biggr)^{\frac{1}{\lambda_{i}}}\neq0. $$

Write

$$\alpha_{i} =\frac{ \vert \omega(x_{1},x_{2},x_{3}) \vert ^{\frac {1}{\lambda_{i}}} \vert f_{i}(x_{1},x_{2},x_{3}) \vert }{ (\int_{a_{1}}^{b_{1}}\int_{a_{2}}^{b_{2}}\int_{a_{3}}^{b_{3}} \vert \omega(x_{1},x_{2},x_{3}) \vert \vert f_{i}(x_{1},x_{2},x_{3}) \vert ^{\lambda_{i}}\diamond_{\alpha} x_{1}\diamond_{\alpha} x_{2}\diamond_{\alpha} x_{3} )^{\frac{1}{\lambda_{i}}}} $$

for $i=1,2,\ldots,m$.

From *AG* inequality (11) we have

$$\begin{aligned} & \int_{a_{1}}^{b_{1}} \int_{a_{2}}^{b_{2}} \int_{a_{3}}^{b_{3}}\prod_{i=1}^{m} \alpha_{i} \diamond_{\alpha} x_{1} \diamond_{\alpha} x_{2}\diamond_{\alpha} x_{3} \\ &\quad\leq \int_{a_{1}}^{b_{1}} \int_{a_{2}}^{b_{2}} \int_{a_{3}}^{b_{3}} \Biggl(\sum _{i=1}^{m}\frac{\alpha_{i}^{\lambda_{i}}}{\lambda_{i}} \Biggr) \diamond_{\alpha} x_{1}\diamond_{\alpha} x_{2} \diamond_{\alpha} x_{3} \\ &\quad=\sum_{i=1}^{m}\frac{1}{\lambda_{i}} \int_{a_{1}}^{b_{1}} \int _{a_{2}}^{b_{2}} \int_{a_{3}}^{b_{3}}\biggl( \frac{ \vert \omega(x_{1},x_{2},x_{3}) \vert }{ \int_{a_{1}}^{b_{1}}\int_{a_{2}}^{b_{2}}\int_{a_{3}}^{b_{3}} \vert \omega(x_{1},x_{2},x_{3}) \vert \vert f_{i}(x_{1},x_{2},x_{3}) \vert ^{\lambda_{i}}\diamond_{\alpha} x_{1}\diamond_{\alpha} x_{2}\diamond_{\alpha} x_{3}} \\ &\qquad{}\times \bigl\vert f_{i}(x_{1},x_{2},x_{3}) \bigr\vert ^{\lambda_{i}}\biggr)\diamond_{\alpha} x_{1}\diamond_{\alpha} x_{2} \diamond_{\alpha} x_{3} \\ &\quad=\sum_{i=1}^{m}\frac{1}{\lambda_{i}}=1. \end{aligned}$$

Therefore, we get the desired inequality (21).

Case (II). By the same method as in Case (I) and using the reversed inequality (11), we can obtain the desired result. □

### Theorem 2.10

*Suppose that* $\mathbb{T}$ *is a time scale*, $a_{i},b_{i}\in\mathbb{T}$ *with* $a_{i}< b_{i}$ $(i=1,2,3)$, *and that* $f_{i}(x_{1},x_{2},x_{3})$, $\omega(x_{1},x_{2},x_{3}): [a_{i},b_{i}]_{\mathbb{T}}^{3}\rightarrow [0, +\infty)$ $(i=1,2,\ldots,m)$ *are* $\diamond_{\alpha}$-*integrable functions with* $\int_{a_{1}}^{b_{1}}\int_{a_{2}}^{b_{2}}\int _{a_{3}}^{b_{3}}\omega(x_{1},x_{2},x_{3})\diamond_{\alpha} x_{1}\diamond _{\alpha} x_{2}\diamond_{\alpha} x_{3}=1$. *Let* $0<\lambda_{1}$, $\lambda _{2}, \ldots, \lambda_{m}<1$ *be such that* $\lambda_{1}+\lambda _{2}+\cdots+\lambda_{m}=k<1$. *Then*

22 $$\begin{aligned} & \int_{a_{1}}^{b_{1}} \int_{a_{2}}^{b_{2}} \int_{a_{3}}^{b_{3}}\omega (x_{1},x_{2},x_{3}) \Biggl(\prod_{i=1}^{m}f_{i}^{\lambda_{i}}(x_{1},x_{2},x_{3}) \Biggr)\diamond_{\alpha} x_{1}\diamond_{\alpha} x_{2}\diamond_{\alpha} x_{3} \\ &\quad \leq\prod_{i=1}^{m} \biggl( \int_{a_{1}}^{b_{1}} \int_{a_{2}}^{b_{2}} \int _{a_{3}}^{b_{3}}\omega(x_{1},x_{2},x_{3})f_{i}(x_{1},x_{2},x_{3}) \diamond_{\alpha} x_{1}\diamond_{\alpha} x_{2} \diamond_{\alpha} x_{3} \biggr)^{\lambda_{i}}. \end{aligned}$$

### Proof

Denote $\xi_{i}=\frac{\lambda_{i}}{k}$ $(i=1,2,\ldots,m)$. Then

$$\xi_{1}+\xi_{2}+\cdots+\xi_{m}=1. $$

Write

$$\psi_{i}(x_{1},x_{2},x_{3})=f_{i}^{k}(x_{1},x_{2},x_{3}) \quad (i=1,2,\ldots,m). $$

From Theorem 2.9 and Lemma 2.5 we have

23 $$\begin{aligned} & \int_{a_{1}}^{b_{1}} \int_{a_{2}}^{b_{2}} \int_{a_{3}}^{b_{3}}\omega (x_{1},x_{2},x_{3}) \prod_{i=1}^{m}f_{i}^{\lambda_{i}}(x_{1},x_{2},x_{3}) \diamond_{\alpha} x_{1}\diamond_{\alpha} x_{2} \diamond_{\alpha} x_{3} \\ &\quad= \int_{a_{1}}^{b_{1}} \int_{a_{2}}^{b_{2}} \int_{a_{3}}^{b_{3}}\omega (x_{1},x_{2},x_{3}) \prod_{i=1}^{m}\psi_{i}^{\xi_{i}}(x_{1},x_{2},x_{3}) \diamond_{\alpha} x_{1}\diamond_{\alpha} x_{2} \diamond_{\alpha} x_{3} \\ &\quad\leq\prod_{i=1}^{m} \biggl( \int_{a_{1}}^{b_{1}} \int_{a_{2}}^{b_{2}} \int _{a_{3}}^{b_{3}} \omega(x_{1},x_{2},x_{3}) \psi_{i}(x_{1},x_{2},x_{3}) \diamond_{\alpha} x_{1}\diamond_{\alpha} x_{2} \diamond_{\alpha} x_{3} \biggr)^{\xi_{i}} \\ &\quad=\prod_{i=1}^{m} \biggl( \int_{a_{1}}^{b_{1}} \int_{a_{2}}^{b_{2}} \int _{a_{3}}^{b_{3}}\omega(x_{1},x_{2},x_{3})f_{i}^{k}(x_{1},x_{2},x_{3}) \diamond_{\alpha} x_{1}\diamond_{\alpha} x_{2} \diamond_{\alpha} x_{3} \biggr)^{\frac{\lambda_{i}}{k}} \\ &\quad\leq\prod_{i=1}^{m} \biggl( \int_{a_{1}}^{b_{1}} \int_{a_{2}}^{b_{2}} \int _{a_{3}}^{b_{3}}\omega(x_{1},x_{2},x_{3})f_{i}(x_{1},x_{2},x_{3}) \diamond_{\alpha} x_{1}\diamond_{\alpha} x_{2} \diamond_{\alpha} x_{3} \biggr)^{\lambda_{i}} \end{aligned}$$

for $k<1$. Therefore the proof of Theorem 2.10 is completed. □

Let $f_{i}^{\lambda_{i}}(x_{1},x_{2},x_{3})$=$\mu _{i}(x_{1},x_{2},x_{3})$, that is, $f_{i}(x_{1},x_{2},x_{3})$=$\mu _{i}^{\frac{1}{\lambda_{i}}}(x_{1},x_{2},x_{3})$ for $i=1,2,\ldots,m$. Then from Theorem 2.10 we get the following Hölder-type inequality.

### Corollary 2.11

*Suppose that* $\mathbb{T}$ *is a time scale*, $a_{i},b_{i}\in\mathbb{T}$ *with* $a_{i}< b_{i}$ $(i=1,2,3)$, *and that* $g_{i}(x_{1},x_{2},x_{3})$, $\omega(x_{1},x_{2},x_{3}): [a_{i},b_{i}]_{\mathbb{T}}^{3}\rightarrow [0, +\infty)$ $(i=1,2,\ldots,m)$ *are* $\diamond_{\alpha}$-*integrable functions with* $\int_{a_{1}}^{b_{1}}\int_{a_{2}}^{b_{2}}\int _{a_{3}}^{b_{3}}\omega(x_{1},x_{2},x_{3})\diamond_{\alpha} x_{1}\diamond _{\alpha} x_{2}\diamond_{\alpha} x_{3}=1$. *Let* $0<\lambda_{1}$, $\lambda _{2}, \ldots, \lambda_{m}<1$ *be such that* $\lambda_{1}+\lambda _{2}+\cdots+\lambda_{m}=k<1$. *Then*

24 $$\begin{aligned} & \int_{a_{1}}^{b_{1}} \int_{a_{2}}^{b_{2}} \int_{a_{3}}^{b_{3}}\omega (x_{1},x_{2},x_{3}) \Biggl(\prod_{i=1}^{m}g_{i}(x_{1},x_{2},x_{3}) \Biggr)\diamond_{\alpha} x_{1}\diamond_{\alpha} x_{2}\diamond_{\alpha} x_{3} \\ &\quad\leq\prod_{i=1}^{m} \biggl( \int_{a_{1}}^{b_{1}} \int_{a_{2}}^{b_{2}} \int_{a_{3}}^{b_{3}}\omega (x_{1},x_{2},x_{3})g_{i}^{\frac{1}{\lambda_{i}}}(x_{1},x_{2},x_{3}) \diamond_{\alpha} x_{1}\diamond_{\alpha} x_{2} \diamond_{\alpha} x_{3} \biggr)^{\lambda_{i}}. \end{aligned}$$

## Application

In this section, using the obtained results, we give the following generalization of the Minkowski inequality for the triple Diamond-Alpha integral on time scales.

### Theorem 3.1

*Suppose that* $\mathbb{T}$ *is a time scale*, $a_{i},b_{i}\in\mathbb{T}$ *with* $a_{i}< b_{i}$ $(i=1,2,3)$, *and that* $f_{i}(x_{1},x_{2},x_{3})\ (i=1,2,\ldots,m)$ *and* $\omega(x_{1},x_{2},x_{3}): [a_{i},b_{i}]_{\mathbb{T}}^{3}\rightarrow \mathbb{R}$ *are* $\diamond_{\alpha}$-*integrable functions*.

(I) *If* $p>1$, *then*
  25 $$\begin{aligned} & \Biggl[ \int_{a_{1}}^{b_{1}} \int_{a_{2}}^{b_{2}} \int_{a_{3}}^{b_{3}} \Biggl\vert \omega(x_{1},x_{2},x_{3}) \Biggl(\sum_{i=1}^{m}f_{i}(x_{1},x_{2},x_{3}) \Biggr)^{p} \Biggr\vert \diamond_{\alpha} x_{1} \diamond_{\alpha} x_{2}\diamond_{\alpha} x_{3} \Biggr]^{\frac{1}{p}} \\ &\quad\leq\sum_{i=1}^{m} \biggl( \int_{a_{1}}^{b_{1}} \int_{a_{2}}^{b_{2}} \int _{a_{3}}^{b_{3}} \bigl\vert \omega(x_{1},x_{2},x_{3}) \bigr\vert \bigl\vert f_{i}(x_{1},x_{2},x_{3}) \bigr\vert ^{p}\diamond_{\alpha} x_{1} \diamond_{\alpha} x_{2}\diamond _{\alpha} x_{3} \biggr)^{\frac{1}{p}}. \end{aligned}$$
(II) *If* $0< p<1$, *then inequality* (25) *is reversed*.

### Proof

We prove only case (I). Write $\Phi(x_{1},x_{2},x_{3})=\sum_{i=1}^{m}f_{i}(x_{1},x_{2},x_{3})$. Without loss of generality, we may assume that $\int_{a_{1}}^{b_{1}}\int_{a_{2}}^{b_{2}}\int _{a_{3}}^{b_{3}}\omega(x_{1},x_{2},x_{3})\Phi ^{p}(x_{1},x_{2},x_{3})\diamond_{\alpha} x_{1}\diamond_{\alpha} x_{2}\diamond_{\alpha} x_{3}\neq0$. From inequality (14) for $p,q>0$ and $\frac{1}{p}+\frac {1}{q}=1$ we have

$$\begin{aligned} & \int_{a_{1}}^{b_{1}} \int_{a_{2}}^{b_{2}} \int_{a_{3}}^{b_{3}} \bigl\vert f_{i}(x_{1},x_{2},x_{3}) \omega(x_{1},x_{2},x_{3})\Phi^{p-1}(x_{1},x_{2},x_{3}) \bigr\vert \diamond_{\alpha} x_{1}\diamond_{\alpha} x_{2}\diamond _{\alpha} x_{3} \\ &\quad\leq \biggl( \int_{a_{1}}^{b_{1}} \int_{a_{2}}^{b_{2}} \int _{a_{3}}^{b_{3}} \bigl\vert \omega(x_{1},x_{2},x_{3}) \bigr\vert \bigl\vert f_{i}(x_{1},x_{2},x_{3}) \bigr\vert ^{p}\diamond_{\alpha} x_{1} \diamond_{\alpha} x_{2}\diamond _{\alpha} x_{3} \biggr)^{\frac{1}{p}} \\ &\qquad{} \times \biggl( \int_{a_{1}}^{b_{1}} \int_{a_{2}}^{b_{2}} \int_{a_{3}}^{b_{3}} \bigl\vert \omega(x_{1},x_{2},x_{3}) \bigr\vert \bigl\vert \Phi(x_{1},x_{2},x_{3}) \bigr\vert ^{(p-1)q}\diamond_{\alpha} x_{1} \diamond_{\alpha} x_{2}\diamond_{\alpha} x_{3} \biggr)^{\frac{1}{q}} \\ &\quad= \biggl( \int_{a_{1}}^{b_{1}} \int_{a_{2}}^{b_{2}} \int _{a_{3}}^{b_{3}} \bigl\vert \omega(x_{1},x_{2},x_{3}) \bigr\vert \bigl\vert f_{i}(x_{1},x_{2},x_{3}) \bigr\vert ^{p}\diamond_{\alpha} x_{1} \diamond_{\alpha} x_{2}\diamond _{\alpha} x_{3} \biggr)^{\frac{1}{p}} \\ & \qquad{}\times \biggl( \int_{a_{1}}^{b_{1}} \int_{a_{2}}^{b_{2}} \int_{a_{3}}^{b_{3}} \bigl\vert \omega(x_{1},x_{2},x_{3}) \bigr\vert \bigl\vert \Phi(x_{1},x_{2},x_{3}) \bigr\vert ^{p}\diamond_{\alpha} x_{1} \diamond_{\alpha} x_{2}\diamond _{\alpha} x_{3} \biggr)^{\frac{1}{q}}. \end{aligned}$$

Therefore we get

$$\begin{aligned} & \int_{a_{1}}^{b_{1}} \int_{a_{2}}^{b_{2}} \int_{a_{3}}^{b_{3}} \bigl\vert \omega(x_{1},x_{2},x_{3}) \Phi^{p}(x_{1},x_{2},x_{3}) \bigr\vert \diamond_{\alpha} x_{1}\diamond_{\alpha} x_{2}\diamond _{\alpha} x_{3} \\ &\quad= \int_{a_{1}}^{b_{1}} \int_{a_{2}}^{b_{2}} \int_{a_{3}}^{b_{3}} \bigl\vert \omega(x_{1},x_{2},x_{3}) \Phi(x_{1},x_{2},x_{3})\Phi ^{p-1}(x_{1},x_{2},x_{3}) \bigr\vert \diamond_{\alpha} x_{1}\diamond_{\alpha} x_{2}\diamond _{\alpha} x_{3} \\ &\quad= \int_{a_{1}}^{b_{1}} \int_{a_{2}}^{b_{2}} \int_{a_{3}}^{b_{3}} \bigl\vert \omega(x_{1},x_{2},x_{3})f_{1}(x_{1},x_{2},x_{3}) \Phi ^{p-1}(x_{1},x_{2},x_{3}) \bigr\vert \diamond_{\alpha} x_{1}\diamond_{\alpha} x_{2}\diamond _{\alpha} x_{3} \\ &\qquad{} +\cdots+ \int_{a_{1}}^{b_{1}} \int_{a_{2}}^{b_{2}} \int_{a_{3}}^{b_{3}} \bigl\vert \omega(x_{1},x_{2},x_{3})f_{m}(x_{1},x_{2},x_{3}) \Phi^{p-1}(x_{1},x_{2},x_{3}) \bigr\vert \diamond_{\alpha} x_{1}\diamond_{\alpha} x_{2}\diamond _{\alpha} x_{3} \\ &\quad\leq \biggl( \int_{a_{1}}^{b_{1}} \int_{a_{2}}^{b_{2}} \int_{a_{3}}^{b_{3}} \bigl\vert \omega(x_{1},x_{2},x_{3}) \bigr\vert \bigl\vert \Phi(x_{1},x_{2},x_{3}) \bigr\vert ^{p}\diamond_{\alpha} x_{1} \diamond_{\alpha} x_{2}\diamond _{\alpha} x_{3} \biggr)^{\frac{1}{q}} \\ & \qquad{}\times \Biggl[\sum_{i=1}^{m} \biggl( \int_{a_{1}}^{b_{1}} \int_{a_{2}}^{b_{2}} \int _{a_{3}}^{b_{3}} \bigl\vert \omega(x_{1},x_{2},x_{3}) \bigr\vert \bigl\vert f_{i}(x_{1},x_{2},x_{3}) \bigr\vert ^{p}\diamond_{\alpha} x_{1} \diamond_{\alpha} x_{2}\diamond _{\alpha} x_{3} \biggr)^{\frac{1}{p}} \Biggr]. \end{aligned}$$

Thus we obtain the desired inequality (25). □

## Conclusions

As is well known, the Hölder inequality and its various extensions play a very important role in mathematical analysis. In this paper, based on the definition of the triple Diamond-Alpha integral for functions of three variables, we have presented the Hölder and reverse Hölder inequalities for the triple Diamond-Alpha integral on time scales. Moreover, we gave some new generalizations of the Hölder and reverse Hölder inequalities for the triple Diamond-Alpha integral. Finally, using the obtained results, we have obtained a new generalization of the Minkowski inequality for the triple Diamond-Alpha integral on time scales. In the future research, we will continue to explore other inequalities for the triple Diamond-Alpha integral on time scales.
